# Witnessing and imagination: Proust, trauma, and the expansion of consciousness

**DOI:** 10.3389/fpsyg.2026.1683934

**Published:** 2026-07-29

**Authors:** Lawrence Fischman

**Affiliations:** Department of Psychiatry, Tufts University School of Medicine, Maine Track Program, Portland, ME, United States

**Keywords:** Proust, dissociation, trauma, witnessing, imagination, coming alive, dreams, psychedelics

## Abstract

I treat Proust’s *Á la Recherche du Temps Perdu* as a fictional case history centered around dynamic tension between dissociation, memory, and creativity. I argue that three pivotal breakthroughs of long dissociated memories and feelings in Proust’s novel result from thawing within a dyadically-determined dissociative process, not simply the chance triggering of involuntary memories as the narrator supposes. Dissociation and its thawing are states of consciousness guided by the same dynamics that enable witnessing to transcend dissociation in the context of trauma. Witnessing allows experience to “come alive.” Weaving together elements of Tronick’s dyadic expansion of consciousness, implicit relational knowing, moments of meeting, transitional phenomena, Modell’s ideas on affect and metaphor, Donnel Stern’s writings on *Nachträglichkeit* and the intersubjective field, and observations from dreams and psychedelic states, I mine Proust’s text to claim that imagination is constrained by what we intersubjectively imagine another knowing or reacting to. Conversely, what we cannot imagine another knowing remains unknowable.

## Introduction

It is now widely accepted that trauma can cause dissociation, or a constriction of consciousness in which aspects of experience associated with trauma become inaccessible or “frozen,” yet still influence mood and behavior (van der Kolk and Fisler, 1995; Modell, 2003, 2009; Meyers, 2012; Blum, 2013). It is also generally accepted that witnessing can be useful in breaking through dissociation (Felman and Laub, 1992; Stern, 2009, 2012, 2017, 2022). In this paper, I treat Proust’s *À la Recherche du Temps Perdu* as a narrative history about dissociation in relation to real and imagined loss. I claim that three pivotal breakthroughs in the novel of long dissociated memories and feelings may be understood as a thawing in the dissociative process, not simply the chance triggering of involuntary memories as the narrator supposes. Both dissociation and its thawing are understood as dyadically determined states of consciousness guided by the same dynamics that enable witnessing to transcend dissociation in the context of trauma. I discuss how a witness allows experience to “come alive” in art and in psychotherapy, building to a claim that what we can imagine is constrained by what we can imagine another knowing or reacting to. In developing this account, I apply to Proust’s rich narrative elements of Tronick’s dyadic expansion of consciousness, psychoanalytic models of implicit relational knowing and moments of meeting, Stern’s views of *Nachträglichkeit* and *kairos*, Winnicott’s transitional phenomena, and observations from dreams and psychedelic states.

## The dyadic expansion of consciousness

Tronick asks, “What is it about connectedness that makes it so critical to human experience and to development?” (1998, p. 296). He frames his answer in terms of systems theory principles which drive the intersubjective meeting of minds he calls a “dyadic expansion of consciousness.” An infant and her caregiver are independent, self-organizing systems which create their own states of consciousness. Like all open biological self-organizing systems, each system (individual) tends toward states of increasing coherence and complexity (Sander, 1995). Through a mutually regulated, bidirectional process of recognition, each member of the dyad communicates its subjective experience to the other via highly specific affective and behavioral signals. Critically, increased coherence and complexity depend on each party apprehending what is in the mind of the other, or knowing and feeling known. Miscoordination occurs frequently, but importantly, affords an opportunity for repair and return to coordinated relating (Beebe and Lachmann, 1994). An analogous process of miscoordination and repair between patient and analyst in the therapeutic situation leads to “now moments” and “moments of meeting,” terms for coming to know something new about oneself through apprehending the other’s state of consciousness (Lyons-Ruth et al., 1998; Stern et al., 1998; Tronick et al., 1998).

When two individuals achieve a new way of relating to each other and, by extension, to the world through a coordination of subjectivities, the dyadic system of consciousness they form together is more coherent and complex than that which either party could achieve alone. Tronick states, “At this moment of forming a dyadic state of consciousness, and for the duration of its existence, there must be something akin to a powerful experience of fulfillment as one paradoxically becomes larger than oneself” (p. 296). Tronick emphasizes the affective power of this expanded state by referring to his Still-face experiment, in which the mother, in the midst of normal interactive play is instructed to maintain an emotionless face for a short time. The effect is devastating. The infant’s profound emotional dysregulation resulting from the sudden withdrawal of coordinated consciousness (Tronick et al., 1978; Cohn and Tronick, 1983) is reminiscent of monkeys raised in isolation (Harlow and Zimmermann, 1958) when confronting a feared object. Tronick’s thesis is that for an infant, beginning in the second half-year of life, separation anxiety is “an experience of diminution—literally, a sense of being less coherently organized” (pp. 296–297).

While the notion that two heads are better than one is hardly groundbreaking, what is striking about Tronick’s idea is describing a dyad as a *system of expanded consciousness*, and suggesting that this expansion exerts a “powerful subjective effect” (p. 296) like “fulfillment.” Stern (1985) believed the motivation to share intentions and feelings is powerful enough to warrant consideration as a primary psychological need. What exactly is this subjective feeling of expansion?” How does it relate to imagination?

## The reflected self

As subjects of consciousness dwell within continuously changing internal and external environments, their survival depends upon their accurately assessing and adapting to such changes. In infancy, such adaptation is necessarily a two-person process (Fotopoulou and Tsakiris, 2017). The dyadic state of consciousness discussed above hinges upon each subject generating expectations about the other based on prior experience, against which new experiences are compared. Infants are superb contingency detectors (Beebe et al., 2003; DeCasper and Carstens, 1981; Papousek and Papousek, 1977; Watson, 1994). They sense when another’s response is determined by the infant’s impulse or behavior (contingent), and when the other’s behavior indicates a response to an impulse (a desire, a feeling, an intention, etc.) originating outside the infant’s experience (non-contingent). That is, they know when they are known by another. Contingencies are used to construct models of self-with-other interactions (Stern, 1985; Beebe and Lachmann, 1988).

Contingency detection underpins the process of knowing and being known. Stern identifies contingent responses as the root of one’s sense of authenticity in the psychotherapeutic situation: “When we speak of an ‘authentic’ meeting, we mean communications that reveal a personal aspect of the self that has been evoked in an effective response to another. In turn, it reveals to the other a personal signature, so as to create a new dyadic state specific to the two participants” (1998, p. 917). Contingent affective responses may be the primary means by which we come to know ourselves, and eventually the template for processes of self-reflection. Beyond “I think, therefore I am,” it may be more like: “I know who I am because of how you react to me.” Many writers have affirmed the notion that we see ourselves as we are seen by others (Loewald, 1960; Fonagy et al., 2002; Stern, 2022), Winnicott wrote, “What does the baby see when he or she looks at the mother’s face? I am suggesting that, ordinarily, what the baby sees is himself or herself. In other words the mother is looking at the baby and *what she looks like is related to what she sees there*” (Winnicott, 1971b, p. 112, emphasis in original).

Thus, the powerful subjective sense of expanded consciousness rests upon mutual regulation, which in turn rests upon the discovery that one’s experience can be known and reflected back in the face or behavior of another, that one’s experience is sharable. The quality and depth of this sense of expansion hinges on the perceived absence of defensiveness in mutual regulation. As Stern points out, a moment of meeting can only occur in the psychotherapeutic situation when an interaction results in the spontaneous emergence of an unguarded affective response, derived from what Winnicott (1960/1984) would regard as the analyst’s True Self. The moment of meeting, or in Tronick’s model, the subjective sense of expanded consciousness, emerges from a powerful new affective connection between two momentarily unguarded beings. This new, affectively charged connection changes the implicit procedural rules which govern how to be with that other (Lyons-Ruth et al., 1998; Lyons-Ruth, 1999). The new dimension of self-reflected through another opens up new ways of being with others in general. In effect, it introduces a new way of belonging in the world (Ratcliffe, 2012), and thus *expands the range of what may be imagined*.

## Witnessing and self-reflection

When one speaks of witnessing in the treatment of trauma, one implies an intersubjective process in which one individual experiences some aspect of a traumatic experience as becoming known by another individual. This experience of feeling known allows someone who has been traumatized to see herself through the eyes of another. This new perspective expands one’s view of one’s own experience, constituting a dyadic expansion of consciousness. In this way, feeling witnessed facilitates self-reflection, including the recognition of heretofore dissociated self-states.

If feeling witnessed emerges from a powerful new affective connection between two unguarded beings, what enables one to feel witnessed following trauma, which undermines trust and invokes defense? This is where *nachträglich* effects on memory come into play.

## *Nachträglichkeit* and enigma

The term *Nachträglichkeit* was coined by Freud to explain how trauma may have a delayed effect in producing symptoms. It refers to the way new experiences can change the way one feels about earlier experiences, by supplying a new context within which their prior meaning is reconfigured. In the case of Emma (Freud, 1895, pp. 353–359), a genital molestation enacted through her clothing at age 8 by a shopkeeper did not cause symptoms until age 12, shortly after puberty, when two shop-assistants, one of whom she found physically pleasing, laughed derisively at her clothing. The later incident, experienced through the prism of puberty, cast the earlier incident in a sexualized light, thereafter resulting in Emma refusing to enter shops by herself. While Freud only rarely referred to this term (translated most often by Strachey as “deferred action”) after abandoning his “seduction theory,” Lacan (1953/1954) recognized in Freud’s discussion of the Wolf Man case (Freud, 1918) a novel view of temporality to which he applied the term *après-coup*. Laplanche, 1992/1998, who suggested the term *afterwardsness* (1992/1998), imbricated the concept within a general psychoanalytic model of trauma, unconscious, and interpersonal processes. Laplanche holds that the infant’s unconscious derives from a sexually enigmatic message implanted into her from the caregiver’s unconscious. It is experienced as a foreign body “’stuck’ in the envelope of the ego like a splinter in the skin” (Laplanche, 1998, p. 209).^1^ This implantation, even though passed unknowingly to the infant through the caregiver’s unconscious, is experienced as an enigmatic communication from the caregiver. It presses for meaning-making or mastery (Laplanche, 2003/2011). Saketopoulou (2023, p. 41) explains: “The ego is established as an apparatus that constructs meaning (symbols, fantasies, etc.) and that binds (organizes) enigma.” According to Laplanche, it is precisely our lifelong quest to understand or “translate” this enigmatic message that makes us human.

New encounters carry new enigmas from the unconscious of the other, which become affordances for translating new or re-translating old enigmatic messages. The *nachträglich* process by which present experiences re-translate or alter the meaning of prior ones is inherently relational. Somewhat counter-intuitively, the road to self-reflection travels through the imagined mind of another. Enigma, which initiates the quest for meaning, originates in interpersonal communication, and evolves through subsequent interpersonal encounters. In this sense, imaginal efforts to make new meaning are relational in nature. Translation and re-translation depend upon integrating the perspective of another, as in: “What I think is your ‘message’ makes me see this differently.” Husserl (1989) incorporates this intersubjective process in his theory of perception. According to Husserl, when we view an object from one angle, we create our understanding of the whole object by imagining how someone might see it from another angle. As Fuchs, 2008 puts it, “the Other is always already copresent in my habitual way of perceiving objects” (2008, p. 282). Intersubjectivity is thus an implicit component of conscious and unconscious perception. The process of decoding meanings (of events, stories, etc.) rests upon a similar process of integrating the imagined perspectives of other subjects.

## *Nachträglichkeit* and unpreparedness

Beard (2020) identifies the belated or *nachträglich* occurrence of trauma in her discussion of temporality in Proust’s *À la Recherche*. She cites Caruth and Rottenberg in emphasizing trauma as an initially “missed event,” an event occurring within a constricted state of consciousness. Caruth views consciousness as “a barrier of sensation and knowledge that protects the organism by placing stimulation within an ordered experience of time” (1996, p. 61). Trauma causes a “break in the mind’s experience of time” (p. 61). It is a response to an overwhelming event or events which initially defies comprehension and expresses itself in delayed, repetitious, involuntary, intrusive symptoms. The full experience of the wound is not known or comprehended at the moment it occurs, “because it is not directly available to experience” (p. 61). The witnessing of the event takes place indirectly and over time, through the involuntary triggering of memory by chance, as happened with Emma when the assistant shopkeepers laughed at her clothing. Caruth emphasizes “that the most direct seeing of a violent event may occur as an absolute inability to know it; that immediacy, paradoxically, may take the form of belatedness” (pp. 91-92). This belated response incorporates a sense of not knowing or unpreparedness derived from “the fact that the threat is recognized as such by the mind one moment too late…. The shock of the mind’s relation to the threat of death is thus not the direct experience of the threat, but precisely the missing of this experience, the fact that, not being experienced in time, it has not yet been fully known” (p. 62).

Rottenberg holds that in *Beyond the Pleasure Principle*, “when Freud defines trauma in terms of ‘the factor of surprise [das Moment der Überraschung]’ ‘the element of fright,’ and the ‘lack of any preparedness for anxiety’^2^, his model of trauma is no longer quantitative but temporal” (2019, p. 32). Rottenberg sees the untimeliness of the traumatic event, which catches consciousness unprepared, as crucial to Freud’s theory of trauma. As Beard (2020) notes, Caruth and Rottenberg both recognize Freud’s shift from an essentially quantitative (too much) view of trauma and the repetition compulsion to a temporal (untimeliness) one in which *Nachträglichkeit*, the afterwards re-shaping of the missed traumatic event by subsequent experience, is indispensable.

## *Nachträglichkeit* and shame

That both authors emphasize the incomprehensibility of the initial trauma is quite compatible with Laplanche’s view of the implantation of the enigmatic message as traumatic. In both accounts, the nature of trauma eludes consciousness. Before discussing Beard’s application of untimeliness and missed experience to Proust, I will mention another view compatible with this accounts of the dynamics of *Nachträglichkeit*.

Green’s (2017) discussion of Lacanian theory and temporality brings together the concepts of *Nachträglichkeit* and shame. For Lacan, the most fundamental question we ask about our origins is ‘What does the other want of me?’ (p. 89). How we interpret what the Other desires of us becomes the “fundamental fantasy” which guides the way we view ourselves. Our sequential encounters with the Real (contingent events, accidents or bad luck) expose “our constitutional lack—the void of non-meaning around which we are constituted” (p. 76) and our lack of mastery and control. Thus exposed, we experience the gaze of the Other as mortifying. But to the extent we can bear this shameful recognition of our helplessness and vulnerability, encounters with the Real also enable us *nachträglich* to re-transcribe the way we view ourselves. In submitting to the shameful reality of our lack, we momentarily escape our fantasy of what the other desires and avoid both “becoming frozen in sterile repetitions” and acting “immodestly without regard for the impact we have on others” (p. 76). We shall see how Proust’s narrator avoids becoming frozen by submitting to his shameful lack below.

Whether it is through unconscious implantation, untimeliness, or a state of overwhelm (Saketopoulou, 2023), trauma arises in a state of constricted consciousness. Enigma, unpreparedness, and overwhelm are all ways of characterizing the subject’s unformulated primary reaction to trauma. It may be most useful to think of them collectively as destabilizing affective dimensions of trauma which impel the subject toward dissociation, repression or disavowal. They are also disturbing self-with-other states that, for reasons discussed above, clamor through compulsively repeated enactments and chance interactions for translation and re-translation aimed at feeling less confused, more prepared, less mortified.

When, as in a moment of meeting or a drug-induced disengagement of defenses, such novel re-transcription occurs, it is subjectively experienced as an expansion of consciousness. Shame, the felt reaction to exposure, is a frequent barrier to such moments, which enable a newly exposed state of self to emerge within a self-with-other (dyadic) context.

## *Nachträglichkeit* and the expansion of consciousness: *la petite madeleine*

Beard traces the presence of *Nachträglichkeit* throughout Proust’s novel, particularly in relation to involuntary memory and the processing of the trauma. She writes,

*Nachträglichkeit* is fundamental to an understanding of involuntary memory in Proust: experience is something that is not fully grasped in its original moment, and the operation of involuntary memory is required in order to recuperate or “master” the experience. Involuntary memory can be read as a mode of repetition compulsion, in which the consciousness, which was absent or overwhelmed at the moment of an experience, finally reclaims that experience.

This is illustrated in the famous *petite madeleine* sequence, in which the taste of madeleine crumbs soaked in hot tea suddenly transports the narrator (Marcel)^3^ back to his childhood in Combray.

But at the very instant, when the mouthful of tea mixed with cake crumbs touched my palate, I quivered, attentive to the extraordinary thing that was happening inside me. A delicious pleasure had invaded me, isolated me, without my having any notion as to its cause. It had immediately rendered the vicissitudes of life unimportant to me, its disasters innocuous, its brevity illusory, acting in the same way that love acts, by filling me with a precious essence: or rather this essence, was not merely inside me, it was me. I had ceased to feel mediocre, contingent, mortal (Proust, 1913/2003, p. 45).^4^

The tea’s effect of rendering the “vicissitudes of life unimportant,” “its disasters innocuous” illustrates Proust’s conception of afterwardsness not only as traumatogenic as in Freud, but as *the means of transcending trauma*. This *nachträglich* transcendent experience is repeated in a few other key passages in *À la Recherche*, all of which employ what Beard refers to as involuntary or “contingent” memory, because of its dependence upon chance circumstances. For Proust’s narrator, the “mark of authenticity” of involuntary memories is “that I was not free to choose them, they were given to me, just as they were.… (T)he very fortuity, the inevitability of the manner in which the sensation was encountered, controlled the authenticity of the past that it resuscitated” (Proust, 1927/2003, p. 187). As noted above, in psychotherapy, the sense of authenticity arises from responses which are felt to be both contingent^5^ upon an aspect of oneself *and* stamped with a unique personal signature.

As the last quotation reveals, the narrator’s search for lost time is linked with a search for an authentic past. His aspiration to become a writer is predicated on making creative use of authentic memories, but his voluntary memory lacks some vital quality. As Beard notes, he cannot make creative use of his experience until it is given new meaning by some chance (found) event in the present. It is only through this second event that the narrator’s past, and with it his creativity, is ignited *nachträglich*.

## Marcel’s “missed experience”

*À la Recherche* begins with the line, “For a long time, I went to bed early,” and immediately falls into a dreamy narrative of vivid impressions and overlapping states of consciousness as Marcel drifts in and out of sleep. For delicate Marcel, safe passage into this beautiful but frightening world required a goodnight kiss from his mother. Awaiting this kiss when his mother was entertaining guests was excruciating. One night, after she refused to answer his desperate note, he threw himself in front of her on her way to bed. Instead of punishing him as he expected, his parents felt sorry for him. The narrator writes: “It seemed to me that my mother had just made me a first concession, which must have been painful to her, that this was a first abdication on her part from the ideal she had conceived for me, and that, for the first time she, who was so courageous, had to confess herself beaten” (Proust, 1913/2003, p. 38)—and he recognized this, saw it move her to tears and metaphorically age her. Her staying in bed with him that night was a Pyrrhic victory: “if I had just gained a victory, it was over her, that I had succeeded, as illness, affliction, or age might have done, in relaxing her will, in weakening her judgment, and that this evening was the beginning of a new era, would remain as a sad date” (p. 38).

This leads directly to Marcel’s dissociating a large chunk of his childhood in Combray. In keeping with Caruth’s and Rottenberg’s notion of a *missed experience*, Marcel tells us, “if I had dared, now, I would have said to Mama: ‘No, I don’t want you to do this, don’t sleep here”’ (p. 38), but “now that the harm was done” (p. 39), the opportunity to say this was gone. Ironically, the gentleness he sees in his mother’s face, his mother’s compassion for him, now pains him.

To be sure, my mother’s lovely face still shown with youth that evening, when she so gently held my hands, and tried to stop my tears; but it seemed to me that this was precisely what should not have been, her anger would have saddened me less than this new gentleness, which my childhood had not known before; it seemed to me that with an impious and secret hand, I had just traced in her soul a first wrinkle and caused a first white hair to appear. At the thought of this, my sobs redoubled, and then I saw that Mama, who never let herself give way to any emotion with me, was suddenly overcome by my own, and was trying to suppress a desire to cry (p. 39).

He recognizes that by insisting that his mother comfort him, he has forced her into surrendering the ideal she held out for him. From this point on, seeking gentleness signifies failure, not living up to the Other’s ideal, and must therefore be avoided, until he changes his mind and accepts Mama’s offer of tea, which breaks through his dissociation and allows her warmth and gentleness to flood back in.

## Combray regained

Just prior to his first startling involuntary memory, the narrator says that “‘everything about Combray that was not the theater and drama of my bedtime had ceased to exist for me, when one day in winter, as I returned home, my mother, seeing that I was cold, suggested that, contrary to my habit, I have a little tea. I refused at first, and then, I do not know why, changed my mind” (Proust, 1913/2003, p. 45). What follows is the dramatic breakthrough partly described above.

What I wish to call attention to is what preceded the extraordinary “chance” epiphany, as elements of this are repeated in similar experiences which occur later in the novel. The narrator’s mother saw that he was cold and offered tea. He initially refuses, as was his habit, but then for reasons he says he does not understand, he *changed his mind*, and accepts her offer.

I suggest that his extraordinary experience did not begin with the mouthful of tea, but with his unaccustomed response to his mother’s offer of tea. His mother’s unexpected gentleness and tears on that fateful night had frozen his memory, isolated from it everything else about Combray. My thermal reference is deliberate, as, I think, are Proust’s. The narrator is caught in repeatedly seeking yet avoiding gentleness and warmth. “*Seeing that I was cold*,” his mother offers him hot tea, catches him unaware, breaks through his defensive habit of making do on his own without warmth. In accepting his mother’s offer, he unwittingly evokes the *possibility of receiving warmth from her* which unfolds from his mouthful of hot tea. But he remains oblivious to the source of his expanded consciousness which passes all too quickly. He tries to recapture the moment by taking in another mouthful of tea. He tries 10 times to revive his pleasurable feeling, but each time “the *laziness* that deters us from every difficult task, every work of importance, *has counseled me* to leave it, to drink my tea and think only about my worries of today, my desires for tomorrow” (p. 47; my emphasis). His language suggests his expansion of consciousness is rooted in dyadic forms, in his replacing his struggle to become that which the Other desires—a writer—with a concession to another voice in his head, that of “laziness.” Admittedly,^6^ this is dyadically-inspired only in an abstract sense, but the fact that a similar shift precedes another momentous involuntary memory, as I will discuss below, strengthens this argument.

Immediately upon relinquishing his voluntary efforts, it comes to him. The taste of the madeleine reminds him of the madeleines soaked in tea that his Aunt Léonie gave him in Combray (Proust, 1913/2003, p. 47).^7^ He wonders if seeing, but not tasting, other madeleines over the years caused Leonie’s madeleines to have “lost the force of *expansion* that would have allowed them to rejoin my c*onsciousness*” (p. 47; my emphasis). Not realizing how accepting hot tea from his mother helped expand his consciousness, he views his breakthrough strictly as a result of the confluence in taste between present and past madeleines. His dyadically expanded consciousness resurrects Léonie’s bedroom and old gray house and rapidly expands to incorporate all of Combray, “the town, from morning to night, and in all weathers, the Square, where they sent me before lunch, the streets where I went on errands, the paths we took if the weather was fine” (p. 47).

## A moment of meeting and expanded consciousness: the Princesse de Guermantes’s party

Outside of this momentary breakthrough, his desire to be a writer, like the rest of his existence, lives in the shadow of his failure to embody his mother’s ideal, and becomes a dominant, frustrating motif in his life. This culminates in his train ride back to Paris after several fruitless years in a sanatorium. When the train stops in an open countryside before a line of trees whose upper trunks reflect the sun, he thinks to himself, “Here I am, after all, in the middle of nature, my eyes, noting the line which separates your glowing fully from your shaded trunks, and I feel only coolness^8^ and boredom. If ever I could have thought of myself as a poet, I now know that I am not” (Proust, 1927/2003, p. 163). He returns to Paris without joy or ambition, where he receives an invitation from the Princesse de Guermantes. Seeing the name Guermantes on the card “re-awakened a ray of my attention, which was to lift from the depths of my memory a section of their past, accompanied by all the images of seigneurial forest, and tall flowers which had then accompanied it, and took on again for me, all the magic and significance, which I used to find at Combray” (p. 164). He attributes his expanded consciousness to the magic of the Guermantes name, rather than to a release from his doomed habitual efforts to write. In a car on his way to the party the streets leading to the Champs-Elysees are so familiar that the car seemed to be smoothly gliding over them of its own accord, “because, in fact, I was no longer having to make the effort of adaptation or attention, which we make, without even being aware of it, when we come across something new” (p. 166). This signals a new state of consciousness. “I was not passing through the same streets as those who were out walking along them that day, but instead moving through a shifting past, sad and gentle” (p. 167).

Getting out of the car, he encounters a drastically changed M. de Charlus recovering from a stroke. He learns that Charlus can “now see perfectly well again,” having only temporarily been blind. His “unruly forest” of gleaming silver hair and flowing white beard, lend him the “Shakespearean majesty of a King Lear” (p. 167), a figure more sinned against than sinning. The new Charlus expends great effort to salute Mme de Saint-Euverte, a woman whom the old Charlus had always dismissed as beneath him. The narrator is astonished by this about-face. Witnessing this moment of meeting between Charlus and St. Euverte stirs an analogous process in himself. He compares Charlus’s greeting of Saint-Euverte to “the politeness of a child, coming timidly forward, at his mother’s request, to say how do you do to some important people. And, indeed a child, without a child’s pride, was what he had become” (p. 169). Charlus has done what the narrator cannot do: dispense with habit and regain his childhood consciousness. The child metaphor evokes Marcel’s anguish-filled night as a child in Combray awaiting a chance to kiss his mother. The allusion is extended by the narrator’s impression that age and affliction have relaxed Charlus’s will, just as Marcel’s importunate behavior that night “succeeded, as illness, affliction, or age might have done, in relaxing [his mother’s] will,” (Proust, 1913/2003, p. 38). The narrator concludes, “for myself, I saw something more like physical gentleness, and a detachment from the realities of life, characteristics so marked among those over whom death has already cast its shadow” (p. 168). Charlus’s “new gentleness” has stirred the narrator’s memory of his reaction to his mother’s unexpected gentleness that night.

The narrator sees “two distinct M. de Charluses,” one “intellectual” but failing to contain a “subconscious” one who ingeniously substitutes new words for habitual ones and searches for pleasurable if illicit sexual liasons with young men (pp. 169–170). I suggest reading this encounter with Charlus in the context of the narrator’s *own changing state of consciousness*, recognizing the futility of his writerly habits. Can the narrator, like Charlus, regain lost time, admit gentleness again, create unexpected word associations, see with his childhood consciousness?

Immediately prior to entering the Princesse de Guermantes’s party, the narrator makes a final attempt to extract a “snapshot” from his childhood, but this again fails, confirming his lack of talent for worthy writing. He states, “It is just when everything seems to be lost that we experience a presentiment that may save us; one has knocked on all the doors which lead nowhere, and then, unwittingly, one pushes against the only one through which one may enter, and for which one would have searched in vain for a hundred years, and it opens” (p. 174). In his distracted state, he does not see an approaching car (his lack of vigilance suggests he has relaxed his habitual defensive efforts), and in stepping suddenly out of the way at the chauffer’s shout, he trips over unevenly laid paving stones. As he regains his balance, he sets his foot upon a stone that is slightly lower than its neighbor, which triggers a dramatic change in feeling. Just as when he tasted the madeleine, “all uneasiness about the future, and all intellectual doubt were gone. Those that had assailed me a moment earlier about the reality of my intellectual talent, even the reality of literature, were lifted, as if by enchantment” (p. 175). He suddenly feels as if he is in Venice. The sensation of the uneven paving stones recalls the two uneven flagstones he stood upon in the baptistery of St Marks. Within moments the clink of a spoon against a plate and the starched stiffness of a napkin trigger memories of Balbec with the same surreal vividness. This awakens a “being within me” who enjoyed “something shared between a day in the past and the present moment, something extra- temporal.” By existing “outside of time” this being “lived only through the essence of things” (p. 179), was not concerned with anxieties about death or the future.

The narrator feels “a considerable appetite for living now that a real moment of the past had just, on three separate occasions, been re-created within me” (p. 180). His ideas about what it means to be a writer have metamorphosized again. His earlier doubts about his own talents and the “falsity of literature” (p. 163) are replaced with new enthusiasm, new ideals. “Real life, life finally uncovered and clarified, the only life in consequence lived to the full, is literature” (p. 204).

In both this and the *petite madeleine* episodes, his breakthrough into “involuntary” memory is preceded by repeated futile efforts to probe his memory for authentic impressions only to give up, temporarily releasing him from a habit doomed by its imbrication with possessing his mother’s heart. Both episodes of involuntary memory are also heralded by moments of meeting, contingent interactions which alter the dyadic possibilities in a relationship (his accepting his mother’s offer of tea and Charlus’s stunning reversal of attitude toward St. Euverte.

## Writing and being witnessed

The narrator’s transformation in the last section is often discussed as contingent upon fortuitous events: stepping upon uneven paving stones, the clink of a spoon, the stiffness of a napkin, or as in the earlier example, the taste of the madeleine. The narrator views his transformation in this way. But as I have tried to show, these seemingly fortuitous events occur in an already changed state of consciousness brought about by new relational contingencies, and it may be more accurate to say that because of this changed state of consciousness, the narrator is better able to make creative use of what he “fortuitously” finds. Moreover, we can understand the state of consciousness which conduces to this creative use as expanded through a process analogous to witnessing.

The narrator’s notions about writing, revitalized at the Princesse’s party, are deeply rooted in his sensitivity to pain and pleasure. His acute sensitivity to both, encapsulated in the dazzling but terrifying character of his dreams that opens the novel, determines his desperate need for his mother’s kiss and leads to the bedtime drama which subsequently delimits the range of his consciousness. In a general sense, this drama concerns the management of a sensitive nature and fertile imagination in an often harsh world, a drama with which many readers will identify.

We have seen how the recovery of his lost childhood in Combray involves the *nachträglich* re-working of the original trauma by later events. What may be less clear is the role of witnessing. I suggest that witnessing is embedded in the very structure of the novel, which by and large is given by a narrator sharing his experience with his reader whose attentive presence is always implied. For example, in the statement quoted above, the narrator writes, “It is just when everything seems to be lost that we experience a presentiment that may save us.” The narrator’s innumerable references to “we” and “us” throughout the novel reinforce the idea that he is never writing in a vacuum, but gauging his experience through the reaction of an imagined witness, the reader.

This process of witnessing, or knowing and being known, occurs between the narrator and other characters in the narrative, too. As Marcel anxiously awaits his mother’s response to his brazen note to her while she is entertaining M. Swann, he tells us, “I thought Swann would surely have laughed at the anguish I had just suffered if he had read my letter and guessed its purpose; yet, on the contrary, as I learned later, a similar anguish was the torment of long years of his life and no one, perhaps, could have understood me as well as he” (Proust, 1913/2003, p. 30). The narrator is concerned here, as he is elsewhere with other characters, with how Swann would understand his experience if he knew about it. He is thinking about Swann as his witness.

His theories about life and art, inspired by his “involuntary” memories at the Princesse de Guermantes’s party, are also centered around witnessing.

(Art) is the revelation, which would be impossible by direct or conscious means, of the qualitative difference in the ways we perceive the world, a difference which, if there were no art, would remain the eternal secret of each individual. It is only through art that we can escape from ourselves and know how another person sees a universe which is not the same as our own…. Thanks to art, instead of seeing only a single world, our own, we see it multiplied and have at our disposal as many worlds as there are original artists (Proust, 1927/2003, p. 204).

Art does what witnessing does in the setting of trauma (and elsewhere). Art expands the way one sees the world, which otherwise would remain one’s “secret;” allows one to escape one’s own consciousness by exposing oneself to the creative perception of another. A key component of his theory is that the rendering of personal experience into art necessarily involves imagining the way an observer might understand one’s experience, and that this imagining, which is intersubjective and therefore relational or dyadic, is what allows the artist *to see his own life*. The narrator says, “this art, complicated though it may be, is actually the only way that art is alive. It alone can express for others, and make us see for ourselves, our own lives, lives which are unable to keep a watch on themselves, and whose visible manifestations, such as they are, need to be translated, and frequently to be read against the grain and painstakingly deciphered” (p. 205).

## Expanded consciousness, coming alive, and witnessing

What makes things come alive for Proust’s narrator is the superimposition of imagination with sensory perception, a kind of waking dream.

So many times in the course of my life reality had disappointed me because at the moment when I perceived it, my imagination, which was my only organ for the enjoyment of beauty, could not be applied to it, by virtue of the inevitable law which means that one can imagine only what is absent. But now all the consequences of that iron law had suddenly been neutralized, suspended, by a wonderful natural expedient, which had held out the prospect of a sensation—sound of a fork and a hammer, same book title, etc.—both in the past, which enabled my imagination to enjoy it, and in the present, where the actual shock to my senses of experiencing the sound, the touch of linen, etc., had added to the dreams of the imagination the thing which they were habitually deprived of, the idea of existence—and, thanks to this subterfuge, had allowed me to obtain, to isolate, to immobilize—for the duration of a flash of lightning—the one thing it never apprehends: a little bit of time in its pure state (Proust, 1927/2003, p. 180).

For Proust’s narrator, art, which he equates with “real life” (p. 204) in contrast to “the work carried out by our vanity, our passion, our imitative faculties, our abstract intelligence, our habits,” (p. 205) comes alive when what we imagine coincides with what we discover through our senses, which confers “an idea of existence” to what would otherwise only be a dream.

This fortuitous coinciding of what is created in one’s imagination with what one finds in the external world bears a striking similarity to Winnicott’s (1971e) ideas about transitional phenomena. The belief a child has in the transitional object owes to its paradoxically being both created and found. “In health the infant creates what is in fact lying around waiting to be found. But in health the object is created, not found…. A good object is no good to the infant unless created by the infant. Shall I say, created out of need? Yet the object must be found in order to be created. This has to be accepted as a paradox” (Winnicott, 1963/1984, p. 181). For Proust’s narrator, it is just in that moment when an object is paradoxically both created in one’s imagination and found in the world that the object, or perhaps more accurately its beholder, comes alive.

I suggest that this “coming alive” is virtually identical to the subjective sense of an *expansion of consciousness* as described by Tronick. The dyadic underpinning of the feeling of expanded consciousness points to the implicit relational dimension of “coming alive.”

Where witnessing crucially enters this process of coming alive is in constructing the space in which such phenomena can be experienced. Winnicott states, “An essential part of my formulation of transitional phenomena is that we agree never to make the challenge to the baby: did you create this object, or did you find it conveniently lying around? That is to say, an essential feature of transitional phenomena and objects is a quality in our attitude when we observe them” (Winnicott, 1971c, p. 96). Transitional phenomena in the child or adult are enabled by the attitude of the parent or of the analyst in accepting the paradox. Taking this a step further, the transitional object is alive for the child because the child senses that the parent knows it is alive for her.

This holds equally for the writer/artist. Just as the child’s belief in the sentient existence of the transitional object is contingent upon the parent’s provision of an attitude that recognizes the child’s need to believe, the writer’s sense of the truthfulness of his own impressions is enabled by the reader’s willing suspension of disbelief. The construction of metaphor, the ultimate distillation of the narrator’s theory of art,^9^ is not a solipsistic exercise but one with an implied witness, an Other for whom the relation seen in the juxtaposition of impressions will have personal meaning, even if different from the writer’s. “The writer’s work is only a kind of optical instrument which he offers the reader to enable him to discern what without this book he might not perhaps have seen in himself. The recognition within himself, by the reader, of what the book is saying, is the proof of its truthfulness, and vice versa” (Proust, 1927/2003, p. 220).

Without an implicit field of observation, imagination cannot attain to more than meaningless fantasy. In a case history, Winnicott (1971a) discusses the distinction between what he terms “fantasying” or daydreaming, on the one hand, which occurs in a dissociated state and cannot be used meaningfully in analysis, and “dreaming” and “real living” on the other hand, which involve “object-relating” and which can be used creatively and understood as having meaning. Winnicott provides a listening presence for his patient that contrasts with her experience of her parents in childhood. Over time, her “confidence in her analyst” allows her to move from “formlessness” into “something” (pp. 33–34). She begins to lose her “rigidly organized dissociations” and “at the same time the fantasying is changing into imagination related to dream and reality” (p. 27). The point here is that the active presence of the analyst for the patient, like that of the reader for the writer, brings to life what would otherwise be idle fantasy or empty memory. Creative imagining, mind-wandering that comes alive, implies a witness.

## Trauma, transitional states and “the place where we live”

In the last section, a link was made between the paradox of “created and found” that is the hallmark of transitional states, and the coming alive of one’s imagination, at least the narrator’s imagination in *À la Recherche*. In this section, we will examine more closely what coming alive means with regard to states of consciousness.

When the narrator re-awakens his past through an involuntarily triggered memory, he feels he has regained lost time because he is re-experiencing not just a memory, but a full state of consciousness from the past.

These resurrections of the past, for the second they last, are so complete that they do more than simply force our eyes to stop seeing the room around them in order to look at the railway line bordered with trees or the incoming tide. They force our nostrils to breathe the air of places that are actually far away, our will to choose between the different plans they suggest to us, our whole person to believe itself surrounded by them, or at least to stumble between them and the present locations, in a dizzying uncertainty akin to that which one sometimes experiences through some ineffable vision at the moment of falling asleep” (Proust, 1927/2003, p. 183).

Or more succinctly, “one minute freed from the order of time has recreated in us, in order to feel it, the man freed from the order of time” (p. 181). When the narrator describes such rarified experience with terms such as “real” or “true” or “the essence of things,” it is because he breathes “an air we have breathed before” (pp. 178–179). He momentarily occupies a former state of consciousness and recognizes it unmistakably as *his*. It is as if life is a journey away from oneself, a struggle to regain contact with who one is. But *in an isolated moment outside of time one makes contact through an imagined witness with a self one has lost*.

Winnicott observes that transitional phenomena arise first at the transition between wakefulness and sleep.

All these things I am calling *transitional phenomena.* Also, out of all this (if we study any one infant) there may emerge some thing or some phenomenon—perhaps a bundle of wool or the corner of a blanket or eiderdown, or a word or tune, or a mannerism—that becomes vitally important to the infant for use at the time of going to sleep, and is a defense against anxiety, especially anxiety of depressive type (Winnicott, 1971e, p. 4).

*À la Recherche* begins with the narrator discussing his transitions between waking and sleeping and turns into a seven-volume novel about consciousness and time, focused upon the narrator’s effort to regain the terrible beauty, the transitional state of consciousness in which his childhood impressions were formed. This state of consciousness was built around a deep sense of connection with his mother, which enabled his nightly transition into his dreamworld. Kissing mama was

what I needed so that I could go to sleep happy, with that untroubled peace which no mistress has been able to give me since that time because one doubts them even at the moment one believes in them, and can never possess their hearts as I received in a kiss, my mother’s heart, complete, without the reservation of an afterthought, without the residue of an intention that was not for me (Proust, 1913/2003, p. 189).

After the traumatic goodnight kiss, Marcel loses the vivid impressions he formed along the Méséglise and Guermantes ways (Martens, 2011) and translated into dazzling and terrifying dreams. What attaches to Marcel’s childhood wonder, his imaginative absorption in nature, legend, and romance is a resolve never again to let his need for his mother’s “heart” become so intense. Ironically, in dissociating this need he freezes his memory of Combray, the wellspring of his childhood wonder, leaving an aspiring writer without access to his deepest impressions.

The salient point is that the dynamics of his dissociation and the coming alive of his imagination are dyadically regulated. Only when he is momentarily able to experience his need for the Other’s gentleness and abandon his unfruitful, solipsistic resolve to become a writer, does he find “lost time.”

Just as the attitude of the observer brings alive the transitional object for the child in the inherently traumatic developmental context of separation, so the narrator’s attunement to the reader’s suspension of disbelief enables an expansion of consciousness, allows the narrator to establish an illusion of “essences” existing outside of time. For Winnicott, one’s belief in this illusion is vital; without this there is only compliance or meaninglessness. Like Proust’s narrator, Winnicott views art, the unfettered expression of imagination in play and cultural activities, as “the place where we live” Winnicott (1971a), or “*what life is all about*” (Winnicott, 1971c, p. 98; emphasis in original). “This intermediate area of experience, unchallenged in respect of its belonging to inner or external (shared) reality, constitutes the greater part of the infant’s experience and throughout life is retained in the intense experiencing that belongs to the arts and to religion and to imaginative living, and to creative scientific work” (Winnicott, 1971c, p. 97).

## “Intermittences of the heart” and the role of the witness

We have seen that the narrator’s expansion of consciousness, his renewed sense of purpose in writing, did not follow solely from a (literal or figurative) stumble into involuntary memory, but from changes in a dynamically-determined state of consciousness into which his memories are woven, as a moment of meeting follows a series of now moments (Stern et al., 1998). This allows him to create what was already there for him to find, i.e., an uneven paving stone, the clink of a spoon, a starched napkin, etc. The role of the witness in this dyadic expansion of consciousness, or coming alive, is masterfully developed by Proust in another well-known episode of involuntary memory in the novel.

In this scene, the narrator has returned to Balbec in a misguided, headlong pursuit of an affair with Mme Putbus’s chambermaid. When this does not materialize, he rationalizes that he is hardly disappointed, notwithstanding his having acted “like a veritable madman” (Proust, 1921/2004, p. 151) to find out if the “Giorgione”^10^ were coming, because his new connections would afford him other opportunities. However, on the day of his arrival, in a state of pain he calls “cardiac fatigue,” as he bends down to unlace his boot his whole being is suddenly convulsed by “the tender, concerned, disappointed face of my grandmother, such as she had been on that first evening of our arrival” (p. 155) in Balbec many years before. The memory of her undressing him prompts him to recall “how, an hour before the moment when my grandmother had thus leaned over, in her dressing gown, toward my boots, wandering in the stifling hot street, in front of the *pâtissier*, I had thought I could never, such was the need I had to embrace her, wait for the hour I still had to spend without her” (p. 156).

Now, on returning to Balbec, this involuntary memory of his grandmother is evoked not simply by the kinesthetic memory of bending towards his boot in Balbec, but by an already changing state of consciousness signified by his “cardiac fatigue,” a somatization of the ineffectual emotional state he characterizes in a reverie just before he convulses as an “incapacity to love” (p. 153). His reverie is a reaction to the nonsensical droning of the hotel manager who meets him at the train. When the narrator thanks him for the favor, the manager responds, “’Oh, don’t mention it! It made me waste only an infinite—for ‘infinitesimal’—‘amount of time”’ (p. 154).

Immediately after this exchange: “A convulsion of my entire being” (p. 154). The unconscious conflation of his aching heart with wasting time unlocks his heretofore dissociated grief for his grandmother, and with it the signature of his grandmother’s tender love for him: “an existence, a tenderness,…. a love in which everything so much found in me its complement, its object, its constant direction, that the genius of great men, all the geniuses who may have existed since the world began, would have counted for less with my grandmother than a single one of my faults” (pp. 156–157).

In dissociating the loss of his grandmother, he had excluded the dyadically expanded *way of seeing things* he shared with her. In his new grief, he implicitly acknowledges the role she played in shaping his childhood vision. “I could not bear to look out on those waves that my grandmother had once been able to contemplate for hours on end; the new image of their indifferent beauty was at once completed by the idea that she could not see them” (pp. 160–161). The waves are “indifferent” because he sees them now through her absence: “the luminous plenitude of the beach was hollowing out a void in my heart;^11^ everything seemed to be saying to me, like the paths and the lawns in the public gardens where I had once lost her, when I was very young, ‘We haven’t seen her,’ and beneath the roundness of the pale, heavenly sky, I felt oppressed” (p. 161).

The deeper cause of his “cardiac fatigue,” presaged by *denying his disappointment* over the chambermaid’s absence and the hotel’s manager’s malapropism about lost time, are his “aridity of soul” and “distress and loneliness” (p. 154–155) in relation to serial disappointing affairs of the heart, and his genuine heartache over losing his grandmother. He dreams he is being kept from seeing her, poignantly feels all of the yearning for her he has blocked since her stroke. His dissociation was brought about by condensing the practically unbearable anguish he endured in Balbec waiting to embrace his grandmother with the parallel anguish he endured waiting to kiss his mother in Combray. Amid his newly felt grief he literally mistakes his mother for his grandmother (p. 512). In each of their embraces, he felt the undivided, undistracted love of the other, a sense that he was all the other sought (compare quote from Proust, 1921/2004, pp. 156–157 on p. 18 above with quote from Proust, 1913/2003, p. 189 on p. 17 above); a momentary stay against self-doubt and shame inherent in the enigmatic message.

In finding grief, he loses his grandmother again, but this time in a heartfelt way, prompting the epigram which yields the title of the novel’s chapter: “For to the disturbances of memory are linked the intermittences of the heart” (p. 155). Modell (2003) contends that metaphor, the fount of unconscious creativity, is generated from bodily feelings (more on this below). The narrator’s account supports this claim, as well as my interpretation that the narrator’s “cardiac fatigue” as he bends towards his boot suggests a dyadic state of consciousness already influenced by a yearning for his grandmother/mother’s unwavering love after yet another setback in his fatally flawed pursuit of romance. In opening his heart to grief and acknowledging that romance without love is a waste of precious time, his past experience comes to life, just as it did when he accepted his mother’s warm tea after years of steeling himself against needing her kiss.

## Primary process and metaphor

Before bending down to unlace his boot, the narrator’s access to states of consciousness linked with his grandmother was closed. His subsequent flood of memories and feelings, each stamped with his and his grandmother’s shared vision, is opened by a dim connection between losing time and denying grief made conscious by the hotel manager’s malapropism about wasting time. His acceptance/experience of painful loss expands his state of consciousness despite his grandmother’s physical absence.

In a similar fashion, feeling witnessed enables an expanded *way of seeing* through a shared emotional connection independent of the physical presence of the witness. This sheds light on why the act of writing, whether by a survivor of trauma or by a narrator in a novel about lost time, can sometimes break through dissociation (Richman, 2013). As discussed above, the truthfulness of metaphor, the uniting of two objects through a quality shared by two sensations, is realized in the meaning it may have for another.

Modell claims that metaphor, affect and memory comprise a synergistic system. Within this system, metaphor structures experience by helping us “find the familiar in the unfamiliar” (Modell, 2001). Building on Edelman’s and Freeman’s neuroscientific research about memory being both categorical and re-transcriptive, his account of metaphor fits well with our interpretations of the narrator’s experience in terms of dissociation, losing and finding time again. Modell writes, “In health the metaphoric process helps recontextualize experience. In trauma, experiences are not recontextualized, foreclosing and freezing the metaphoric correspondence between past and present” (Modell, 2001). By frozen, Modell means essentially unavailable for creative use in metaphor, which simultaneously recognizes both similarity and difference. The overwhelming nature of trauma leaves an affective category of such intensity that protectively, with the recognition of similarity, one anticipates that a new event will be identical with a previous traumatic one, foreclosing the play otherwise available in ambiguity, of finding both similarity and difference. In health, metaphor utilizes primary processes that dreams use- condensation, displacement and symbolization- to find the familiar in the unfamiliar, allowing past experience to *come alive with new meaning* through re-contextualization. Modell thus sees metaphor as the basis of *Nachträglichkeit*, or when occurring in a relational context, what I have been describing as a dyadic expansion of consciousness. “In this fashion, trauma will constrict the complexity of consciousness” (Modell, 2001).

Notably, the narrator places his imagined tryst in Balbec, not Paris, where familiarity or habit made it difficult to create the illusion that this encounter would change his life. Habit, he tells us, blinds us to the reality of our impressions. “For, if habit is a second nature, it prevents us from knowing the first, of which it has neither the cruelty, nor the enchantments” (Proust, 1921/2004, p. 153). In this sense, habit, or defense, is the bane of the writer, the antithesis of metaphor. For the reality of his impressions to come alive, something must shake the narrator from habit, as when “contrary to my habit” he accepts his mother’s offer of tea. In that instant, tea became metaphor for his mother’s love, “invaded” him, “acting in the same way that love acts, by filling me with a precious essence: or rather this essence, was not merely inside me, it was me.” The guiding principles of the secondary process, his habits and defenses, gave way. “It had immediately rendered the vicissitudes of life unimportant to me, its disasters innocuous, its brevity illusory.” The primary process enables experience to come alive, “takes two different objects, establishes their relationship” and “by bringing together a quality shared by two sensations… draws out their common essences by uniting them with each other, in order to protect them from the contingencies of time, in a metaphor.”

The primary process in its purest form is most evident and sustained in dreams, to which it lends qualities of presence and immersion (Windt, 2021). It is also evident in psychedelic states (Carhart-Harris and Friston, 2010; Kraehenmann et al., 2017). Modell (2003, 2009) suggests “that *an unconscious metaphoric process, analogous to dreaming, occurs while we are awake*.” We have seen this occur in relation to the narrator’s expanded consciousness in the three occasions discussed above. In the next section we shall see how this unconscious metaphoric process is activated dyadically in a moment of meeting.

## Expansions, contractions, and the interpersonal field

The notion that the expansion of consciousness in dreams and psychedelic states emerges from eliminating defenses is supported by the truth of its converse, that defenses in ordinary states constrict consciousness. This is clearly an adaptive necessity, as our brains cannot attend to everything at once, and must limit their focus to actions which maintain physical and emotional homeostasis and assess potential threats.

The dynamic link between expansion and constriction lies in the regulation of emotion. As Tronick et al. (1998) states:

The expansion of the infant’s state of consciousness emerges from the process of the mutual regulation of emotion. During an interaction, information about the infant’s state of consciousness (e.g., intentions, affects, and arousal level) is conveyed through affective configurations that are apprehended—come to be known—by the mother. In response, the mother provides the infant with regulatory support that permits the infant to achieve a more complex level of brain organization (p. 295).

Because these interactions often go awry (miscoordination) and lead to emotional distress, infants develop implicit “procedures for being with others” that “vary widely along many dimensions, such as in the likelihood of engaging others in positive exchanges, in the affects that are exhibited or not exhibited to others, in the social and affective information that is elicited from others, or in the effectiveness of procedures for eliciting help or comfort from others” (Lyons-Ruth, 1999, p. 590). These implicit procedural models, which govern ways of being with others, guide what kinds of experiences can and cannot be intersubjectively known. Repeated miscoordinations that are unrepaired lead to models with significant “deletions and distortions or ‘incoherencies”’ which “are integral to the developmental origins of some defenses” (Lyons-Ruth, 1999, p. 590).

The fact that interactions leading to implicit procedural models begin well before symbolization and language develop has led infant researchers to a theory of the “dialogic origin of mind,” which holds that the “organization of experience begins as dyadic and dialogic” (Beebe et al., 2003, p. 807); “that *all linguistic forms of intersubjectivity continue to depend on pre- or nonlinguistic forms*” (p. 813; emphasis in original). Regarding intersubjectivity, Stern writes, “What is ultimately at stake is nothing less than discovering what part of the private world of inner experience is shareable and what part falls outside the pale of commonly recognized human experiences” (1985, p. 126). I suggest that what cannot be intersubjectively “known”—to be clear, we are talking mainly about pre-verbal, pre-symbolic experience—cannot be used in the *nachträglich* recontextualization of experience that is creative imagining.

Stern (2017) reaches a parallel conclusion in discussing unformulated experience. In Stern’s view, unformulated experience is potential experience, a “something” (borrowing Laplanche and Pontalis (1967/2018) term) from the past that lacks specific meaning and only acquires significance *nachträglich* through events occurring in the here and now, much as the dissociated past comes to life for Proust’s narrator through involuntary memory. Stern uses the notion of *kairos* in parallel with the way I discuss “imagination” here. In his expanded view of *Nachträglichkeit*, psychological time (*kairos*) floats freely rather than chronologically, constantly linking past and present and reconfiguring each by the other. This continuous creation and recreation of subjectivity is compromised by our need to defend ourselves from the painful, at times threatening, vicissitudes of life. Stern views dissociation as the “primary defensive or self-protective psychic process” (p. 503). Dissociation freezes *kairos*, prevents the spontaneous, creative linking of past and present. “Meaning continues to be created and attributed, but only along familiar, unobstructed paths that, simply because they are so very familiar, have lost their capacity to surprise us, and therefore their vitality” (p. 504).

This is the condition of Proust’s narrator in the wake of his dissociative defenses against the trauma of falling short of his mother’s ideal.

Stern writes, “*The interpersonal field is our route of access to frozen time*” (p. 507; emphasis in original). He illustrates this through his treatment of George, who suffered early traumatic separations that left him feeling like “a fat-thighed piece of shit” (p. 509). As a result, he largely dissociates his desire to be significant to others, including his analyst. At a certain point, with summer vacation approaching and George worrying that he will get depressed during the break, Stern senses that George is on the verge, but is still not quite able to imagine something. He says to George, “Maybe you could imagine that I’ll come back. Maybe you could even imagine that I’ll *want* to come back’. With quiet amazement, George said slowly, drawing out the words, ‘Maybe I *could* think that. Maybe I could”’ (p. 510; emphasis in original).

The exchange enables George to think of himself as someone whom his analyst could look forward to seeing. Stern notes that his interpretation would have fallen flat at another time, but this time he sensed a thawing in George’s dissociation of his feelings toward him. The temperature metaphor here mirrors the temperature metaphors discussed above that presaged the narrator’s breaking through his dissociation after a mouthful of tea-soaked madeleine.

For Stern, *kairos* determines the state of the relational field. When *kairos* is free new meanings are created within the relational field, new ways of being with others are discovered. When *kairos* is frozen, relatednesss is stuck in repetition compulsion mode. While Stern’s emphasis is on time having its own mind (p. 504) and influencing the relational field rather than vice-versa, his therapeutic aim is to thaw the frozen parts of the relational field to release *kairos*. His discussion of how *kairos* determines one’s capacity to use subjectivity in creative living parallels the discussion above of the subject’s capacity to use imagination creatively, to experience a sense of expanded consciousness. In both accounts, eliminating barriers within the relational field enables creative living and feeling alive. When *kairos* is free, so is the imagination. When trauma and dissociation hold sway, imagination and *kairos* are stifled.

The *thawing in dissociation* which guides the timing and effectiveness of Stern’s interpretation is what determines the timing of the narrator’s three breakthroughs of involuntary memory. His loving bonds with his mother and grandmother are built on true gentleness and compassion, but his incessant anxiety about this compels him to strain his relations with them, exaggerate his need, create pain in their faces and then dispel their suffering with his kisses. When he goes too far (with his mother on the anxious, sleepless night in Combray, and with his grandmother when she is dying and insists on having her photograph taken (Proust, 1921/2004, p. 157) he sees his perverseness reflected in their distressed faces. Consequently, he dissociates the intensity of his need, throws himself into his other vain pursuits. Only when his innate vulnerability and gentleness supercede his compulsive need to be all that the other desires does his dissociation thaw, allowing him to re-experience the pleasures he shared with these two great loves. This thawing in the dissociation of a dyadically-based state of consciousness explains why a *petite madeleine*, a bending to unlace a boot, and an uneven paving stone brought his memories to life when they did, and not before.

## Final thoughts

*Nachträglichkeit* was coined initially by Freud to explain the belated appearance of traumatic or hysterical symptoms. It has gradually acquired a wider application in psychoanalysis (Lacan, 1953/1954; Laplanche, 1998; Faimberg, 2007; Dahl, 2010; Goldin, 2016; Stern, 2017; Larsen and Rosenbaum, 2023), so that it may encompass the general way that new experiences alter the meanings of past experiences, and in doing so, alter the subject who experienced them. We have seen that the same mechanism—re-transcription—that gives rise to trauma and dissociation can help to undo it.

Kissing his mother goodnight symbolically offers Marcel a momentary stay against confusion, against “whirling madly through” the “shifting kaleidoscope of the darkness” (Proust, 1913/2003) that comprises his nighttime world. According to Bollas, the infant experiences the mother’s handling or “idiom of care” as a process of transformation, a “profound occasion where the content of the self is formed and transformed by the environment” (Bollas, 1978, p. 386). For the infant, as for Marcel, it is a subjective experience of one’s internal world being given form. Bollas holds that this process of transformation establishes a paradigm for a lifelong search for the transformational object. This account applies well to Marcel’s compulsive pursuit of a romance that will change his life, and a way of writing that will confer a sense of meaning to his memories and impressions. The narrator seems to grasp this concept when he formulates the writer’s relationship with the reader: “The writer’s work is only a kind of optical instrument which he offers the reader to enable him to discern what without this book he might not perhaps have seen in himself. The recognition within himself, by the reader, of what the book is saying, is the proof of its truthfulness, and vice versa” (Proust, 1927/2003, p. 220).

This account of the transformational object dovetails nicely with Tronick’s notion of a dyadic expansion of consciousness, and helps to explain its affective power. I have suggested that this power, felt subjectively as *coming alive*, also owes to the sense of meaningfulness drawn from primary process-derived metaphor, from paradoxically creating and finding, and from the sense of connectedness implicit in knowing and being known. Stern (1985, 2004) posits that the motivation to share intentions and feelings is powerful enough to warrant consideration as a primary psychological need (see also Tomasello et al., 2005). Consider the wonder in recognizing that another being knows just what we need at the very instant we need something.^12^ Coming alive is the re-awakening of that wonder, a forerunner of awe.

I suggest, too, that *what we can create in our imagination coincides with what we can imagine as knowable by another*. Conversely, *what we cannot conceive another person knowing cannot be formulated*. This does not mean we cannot imagine something that no one else has thought of, or something that we would never actually share. It just means that the way we expand consciousness, the way we meaningfully, usefully come to know, is through the imagined perspective of another. Absent that perspective, our neural circuits are canalized, we traverse old ground.

Trauma, “the violent intrusion of something radically unexpected, something the subject was absolutely not ready for, something the subject cannot integrate in any way” (Žižek, 2009, p. 125), is unimaginable and therefore unknowable, a missed experience (Caruth, 1995, 1996; Bistoen et al., 2014; Rottenberg, 2019). Integration awaits imagining a witness who can “know.” This idea of the other “knowing,” rather than sympathizing, validating, re-assuring, etc., is consistent with contemporary relational psychoanalytic conceptions of the role of witnessing in the resolution of trauma (Benjamin, 2016; Davies, 1996; Poland, 2000; Reis, 2009).

Expectancies, the internalized object relations we build from detecting contingencies, enable rapid adaptation and limit surprise (Friston, 2010). But expectancies constrain imagination in the same way that culture constrains language (Everett, 2005). Creative use of imagination requires a surrender of expectancy as occurs in dreams and psychedelic states, in which experience feels unbidden, like the narrator’s involuntary memories. The fleeting candor in a moment of meeting also enables a transient release from contingency and with it, a sense of truthfulness may arise. Campbell-Fisher, 1950 describes how art may afford such a release:

Life has glories of its own, but it is not in daily living that we see patterns freed from, or not yet encumbered by, the contingencies and frustrations of this world of fevered effort…. My grasp of the essence of sadness and its imperishable reality comes not from moments in which I have been sad, but from moments when I have seen sadness before me released from entanglements with contingency (1950, pp. 267–268).

The argument made in this paper suggests that such a release derives from a rapport, a witnessing, a “knowing and being known,” imagined between observer/reader and artist.

In this paper, I have used the fictional experiences of Proust’s narrator as a kind of case history. Reading Lauren Beard’s dissertation inspired me to pick up Proust again. Within a few sentences I was absorbed in the narrator’s world. Interpreting the narrator’s experiences felt no different than interpreting the dreams and experiences of real people. I mention this partly to justify my approach in this paper, and partly because my experience illustrates what I think Winnicott meant in referring to transitional space as “the place where we live” (Winnicott, 1971d).

We are perpetually trying to discern the enigmatic message, trying to figure out what the Other desires of us. Not knowing, the missed experience, is an adaptive liability, a source of shame. Our determination to master the enigma, to know, is often translated into the desire to fulfill our ideals. Our need to know may blind us to what is waiting to be found. The moment we concede and accept our limitations—in the narrator’s case, the moment he admits he lacks the talent to become a writer—we stop seeing mainly what we expect to see, open ourselves to other perspectives. For the narrator, this meant finding time again. In a funny way, for me it meant finding Proust again, to write this paper. I had rummaged through twenty stacked boxes of books (from a recent move) several times over several days searching for two Proust volumes. I “knew” they had to be there. Exasperated, I finally gave up, accepted having to go to the library (i.e., my limitations). I re-stacked the boxes for the last time, peaked inside the last box before closing it, and found Proust again.

## Data Availability

The original contributions presented in this study are included in the article/supplementary material, further inquiries can be directed to the corresponding author.

## Footnotes

1 In the following, my use of Laplanche’s nuanced concept of enigma is limited to the child receiving from the adult caregiver puzzling signs and communications that the child senses are meaningful but cannot yet fully understand.

2 The quotations are from Freud (1920, pp. 12 and 31).

3 When referring to the narrator actively as a child, I will use his name, Marcel.

4 I will discuss the striking similarities between the narrator’s experience in this passage and psychedelic experience below.

5 The word contingent is used differently in literary criticism of Proust and in infant-caregiver research. In the former setting, it refers to the way involuntary memories are evoked by (“contingent” upon) random circumstances arising within the external world. In the latter setting, it refers to the way one subject recognizes another subject’s reaction as related to (contingent upon) the first subject’s behavior. Proust’s narrator feels released from constraints of time when his dissociated childhood memories are evoked by (“contingent” upon) something totally external to his own efforts or experience. I will attempt to show that the narrator’s “involuntary” memories derive organically from immediately antecedent relational interactions, as moments of meeting derive from a build-up of “now moments” (Stern et al., 1998) and are not merely triggered by random environmental circumstances. What bridges Proust’s narrator’s experience with Stern’s view is the idea of the analyst’s “personal signature,” which Stern argues is essential to the moment of meeting. The analyst’s personal signature, like the narrator’s “fortuity,” is totally unrelated to the subject, which lends the subject’s experience a stamp of authenticity (it feels “found” or “given”) instead of feeling forced or contrived.

6 Such abstract dyads, or “partners in thought” (Stern, 2009) are ubiquitous once one is alerted to their forms. Here, the word “admittedly” reveals that I have you, the reader in mind as I write.

7 Aunt Léonie, who is constitutionally similar to the narrator in many ways, voices her impressions aloud to herself, allowing the young narrator continuous access to her state of consciousness in relation to Combray.

8 Note the thermal reference which is again associated with his failure to attain his ideal.

9 “The truth will begin only when the writer takes two different objects, establishes their relationship…. Indeed, just as in life, it begins at the moment, when, by bringing together a quality shared by two sensations, he draws out their common essences by uniting them with each other, in order to protect them from the contingencies of time, in a metaphor” (Proust, 1927/2003, p. 198).

10 The narrator and his friend, Robert de Saint-Loup, longingly refer to the chambermaid as a “Giorgione” after the influential Venetian painter (p. 97).

11 Another cardiac reference.

12 In explaining the value of illusion in the process of disillusionment, Winnicott says, “The mother places the actual breast just there where the infant is ready to create, and at the right moment” (Winnicott, 1971a, p. 11).
